# 

**Published:** 1892-10-01

**Authors:** 


### The Hospital

A Weekly Institutional Journal of

### Science, Medicine, Nursing, and Philanthropy.

EDITED BY

HENRY C. BURDETT,

AUTHOR OF "HOSPITALS AND ASYLUMS OF THE WORLD: THEIR ORIGIN, HISTORY, CONSTRUCTION, ADMINISTRATION, MANAGEMENT AND LEGISLATION"; "HOSPITALS AND THE STATE"; "PAY HOSPITALS OF THE WORLD"; "COTTAGE HOSPITALS, GENERAL, FEVER, AND CONVALESCENT, WITH FIFTY BEDS AND UNDER": "BURDETT'S HOSPITAL ANNUAL AND YEARBOOK OF PHILANTHROPY"; AND "HELPS TO HEALTH."

ACTING EDITORS:

GEORGE W. POTTER, M.D.Edin.,

AUTHOR OF "MINISTERING WOMEN"; AND NUMEROUS PAPERS ON SCIENTIFIC AND MEDICAL SUBJECTS

AND

P. WATSON WILLIAMS, M.D.Lond.,

PHYSICIAN TO THE OUT-PATIENTS AND FOR DISEASES OF THE THROAT, BRISTOL ROYAL INFIRMARY, ETC.

INDEX TO VOL. XIII.

(1st October, 1892, to 25th March, 1893.)

LONDON:

THE SCIENTIFIC PRESS (Limited), 428, STRAND W.C.

1893

W1 H069X

### INDEX TO VOL. XIII. 1892–3.

Aberdeen Hospital for Sick Children, 64
Aberdeen and its Institutions, 29
Aberdeen Royal Infirmary, 40
A Blackburn " Hospital Scandal," 345
A Central Hospital Board for London, 287
A Challenge to the Press, 323
A Clearing House for the Unemployed, 103
A Cry for More Institutions, 386
A Danger to be Averted, 81
Addenbroke Hospital, 56
Adulterated Milk, 136
After Care Association, 200
A Horses' Hospital, 119
Aix-la-Chapelle and its Waters, 108
Alterations in Birmingham Medical Charities, 282
A Masseuses' Secret, 297
An Appeal to Reason, 65
A New Use for Telepathy, 262
Another Hint to the Oxford Infirmary, 322
Anti-Vivisectionists and their Mistakes, 160
" A Plague of Specialists," 393
Artificial Feeding and Food Disorders of Children, 140
Assistants' Manners and Nurses' Fees, 76
Asylums in India :—
I. Bombay Presidency, 292
II. The Madras Presidency, 320
III. The Bengal Presidency, 324
Asylums of New Zealand, 159
Attendants in Olden Times, 127
Attitude of Great Writers Towards Disease (*continued*) :—
XIII. Elizabeth B. Browning, 38
XIV. Sir T. Browne, 70
XV. The Sick in Utopia, 102
XVI. Boccacio
XVII. Charles Kingsley, 148
XVIII. Milton, 164
XIX. Charles Lamb, 180
XX. The Plague at Milan, 1630, 294
Autobiographical, 227
A Wise Resolve, 2
A Word for Poor Physic, 329
Baron de Hirsch's Winnings, 266
Bedford and Its Medical Institutions, 303, 321
Bedfordshire Hospital Trained Nurses' Institution, 321
Bedsteads, 158, 191
Berrywood Asylum, 125
Bitter Cry of Motherhood, The, 33
Bolingbroke House Pay Hospital, 157
Bradford Infirmary, 189
Bridges, Dr., & Metropolitan Asylums Board, 152
Bristol General Hospital, 128
British Home for Incurables, Clapham, 144
British Woman's Temperance Association, 150
" Bromide Habit," The, 281
" Carboline " Disinfectant, 60
Cass's, Sir J., Foundation School, 410
Catholicism and Physiology, 377
Central London Throat and Ear Hospital, 64
Charing Cross Hospital, 346
Charity Organisation Society, 362
Chemistry of Life and Health, The, 366
Cherryhinton to the Rescue 265
Chesterfield and North Derby Hospital, 88
Cheyne Hospital for Sick Children, 214
Children's Cots, 79, 94, 101
Children's Hospital, 128
Children's Hospital, Great Ormond Street, 383
Children's Hospitals, 207
Children's Teeth, The, 409
Choice of an Antiseptic, The, 106
Cholera and Leprosy, 200
Cholera, Birthplace of, 86
Cholera Hospital, Proposed, 362
Cholera One Step Further, 23
Cholera and Opium, 100
Christmas Fare for Children, 223
Christmas Numbers, 198
Christmas Tableaux Vivants, 201
Christmas Thoughts at National Gallery, 191
Chronicle of Fever Hospitals, The, 266
Church of England Sanitary Association, 386
City of London Asylum, 273
City of London Truss Society, 330
Clarence Memorial Wing, St. Mary's Hospital, 200
Colour Blindness, 310
Consumption Hospitals, 206
Contemporary Theory and Research, 326, 360, 392, 406
Converted Clowns as Hospital Chaplains, 87
Cooking by Gas, 239, 255, 272, 287
Cork Hospital and Lady Students, 8
Cost of Trained Nurses, 18
Cremation or Burial, 145
Crinolines, 280
Cumberland Infirmary, Carlisle, 351
Cure of Poverty (*continued*), 15
Dermographism, 278
Dental Hospital, 182
Devon and Exeter Hospital, 46
Diseases of Children, Medical & Surgical, The, 202
Divorce Reform League, [illegible]
Doctors and the Drink Bill, 345
Doctor's Christmas, The, 196
Drinking Women, 248
Diet in Disease :—
I. Food and Food Values, 3
II. Food and Food Values (*continued*), 19
III. Stimulants; Alcohol, 36
IV. Stimulants (*continued*) : Coca of Peru, Coca Wine, 51
V. Restoratives : Tea, 83
VI. Stimulants (*continued*) : Coffee, Cocoa, Chocolate, 116
VII. Water. Salts, 131
VIII. Digestion, 149
IX. Digestion (*continued*), 165
X. Digestion, Absorption, Excretion, 183
XI. Invalid Foods, 213
XII. Diabetes, 311
XIII. Diabetes (*continued*), 343
XIV. Diabetes (*continued*) 359
XV. Diabetes (*continued*), 375
XVI. Diabetes (*continued*), 391
XVII. Gout, 407
Drink at the World's Fair, 152
Droitwich " Home," 56
Drunk or Dying, 55
Dublin Hospital, 120
Dublin Rotunda Hospital, 182
Durham County Hospital, 335
Dwellings for the Poor and the Goldsmiths' Company, 182
Earl Rosebery and the Royal National Hospital for Consumption, Ventnor, 136
Edinburgh Royal Infirmary, 173
Electric Bath Chair, 358
Epilepsy and Paralysis Hospitals, 207
Embryology of Man and Mammals, 318
Employment for Women : IV. Lecturing for County Council, 22
Empyema, 331
Engenolacetic Acid, 310
Enthusiasm of Medicine, 377
Experimental Anti-Choleraic Inoculation, 313
Experiments on Human Subjects, 55
Eyes, 328
1892, 229.
Faces in the Crowd, 241
Faithfulness and Kingship, 113
Fashionable Nerves, 136
Fasson, Dr. H., 191
Feeding of Girls at Schools, The, 243
Feeding of Infants and Children, The :
I. Health, 105
II. In Disease, 122
Fleming Memorial Hospital, 96
Food of " Poor Jack," 313
Foul Language in Public Places, 212
Four Years of Margarine, 151
Frog Farming, 296
Fruits of Vivisection, 67
General Civil Hospital, Colombo, Ceylon, 320
General Infirmary, Northampton, 271
Glasgow Infirmary, 24
Glasgow and Medical Institutions, 93
Great Ormond Hospital for Children, 48
Health of India, The, 310
Health Minister, Proposed, 248
Heart Disease, 87
Heathdene Convalescent Home, 8
Hewer, Mr. J. H., 313
Hirsch, Baron, Donations, 330
Hirsch Baron, Turf Winnings, 370
Hæmorrhoids, 235
Holloway Sanatorium, 273
Home for the Dying, The, 72
Home for Little Boys, 152
Home Hospitals Association, The, 404
Home of Peace for the Dying, 55
Honest Anti-Vaccinators, 281
Hospital for Curing Poor at Home, 158
Hospital Administration (*continued*) :—
III. Special Hospitals, Children's, 13; IV. Lying-in for Women, 31; VI. Ophthalmic and Miscellaneous, 45; Royal Frederick's Hospital, Copenhagen, 80; Hospitals and Asylums of the World, 287; Outpost Hospitals in Large Towns, 399
Hospitals Association, The, 152, 182
Hospitals Association, Progress & Policy of, 275
Hospital and Staff Liability for Negligence, 403
Hospitals and Asylums of the World, 287
Hospital Clinic—
Treatment of Acute Rheumatism, 171, 397
Treatment of the Ankle, Sprains of, 59
Treatment of the Bowel in Hernia, 301, 380
Treatment of Bronchitis, 364
Treatment of Burns and Scalds, 57
Treatment of Cholera, 187, 267, 395
Treatment of Chronic Constipation, 25
Treatment of Chronic Ulcers of the Leg, 106
Treatment of Chronic Suppuration of the Middle Ear, 91
Treatment of Colles's Fracture, 138
Treatment of Conjunctivitis, 332, 413
Treatment of Corneal Ulcers, 41
Treatment of Delatation of Stomach, 283
Treatment of Diphtheria, 42
Treatment of the Dropsy of Bright's Disease, 11
Treatment of Enteric Fever, 89, 155
Treatment of Flat Foot, 73
Hospital Clinic (*continued*)—
Treatment of Fractures of the Femur, 380
Treatment of Fractures of the Leg, 90
Treatment of Fractured Patella, 9, 75
Treatment of Gastric Ulcer, 253, 347
Treatment of Glanular Lids, 121
Treatment of Headache, 396
Treatment of Heart Disease, 221
Treatment of Hernia, 229, 301
Treatment of Hip Disease, 284, 285
Treatment of Knock Knee, 268
Treatment of Injuries to the Hand, 219
Treatment of Intussusception, 395
Treatment of the Middle Ear, 154, 186
Treatment of Morbus Coxae, 315
Treatment of Naevus, 365
Treatment, Operative, of Mucous Polypi of the Nose, 334
Treatment of Otoripsea, 348
Treatment of Pleurisy, 121, 381, 411
Treatment of Pneumonia, 269, 411
Treatment of Post Nasal Adenoides, 139
Treatment of Pott's Fracture, 285, 299
Treatment of Rickets, 185, 347, 363
Treatment of Spinal Disease, 153
Treatment of Tonsillitis, 253
Treatment of Tubercular Diseases of the Knee, 137, 220, 251
Treatment of Tuberculous Glands, 269
Treatment of Typhoid Fever, 58, 167, 333
Hospital Dressers' Table, 400
Hospitals in India :—
I. The Bengal Presidency—Calcutta Medical Institute, 240
II. Madras Presidency, 320
Hospitals in New Zealand, 95
Hospital Saturday Fund, 250
Hospitals of the World :—
I. Early European Hospitals, 385
II. The Progress of English Nursing, 401, 416
Hospital Uncharity, 637
Hospital Sunday Fund, 104
How to Catch Rheumatism, 393
Huddersfield Infirmary, 33
Hull Royal Infirmary, 367
Hygiene and Caligraphy, 167
Hypnal or Monachloral Antypirin, 349
Hypnotism and Drunkenness, 276
In the Abstract, 260
Infected Rags, 328
Inflammation, The Worth of, 132
Influenza Once More, 361
Institute of Preventive Medicine, 353
International Congress of Charities, 290
Invertebrate Medicine, 329
Irish Distressed Ladies' Fund, 266
Irish National Consumption Hospital, 72
Is the London School of Medicine a Failure ? 185
Is Science too Egotistical ? 119
Jack Tar's Cookery, 297
" Keep Your Mouth Shut," 246
Keswick Cottage Hospital, 319
Killarney District Asylum, 273
Kitchen Ranges and Stoves, 418
Lambeth Workhouse and Prince of Wales, 346
Leeds General Infirmary, 77
Lifeboat Saturday Council at Manchester, 346
Light in a Sick Room, 376
Literary Intelligence, 257
Lockers, 368
London Homœopathic Hospital, 43
London Hospital, 354
Longmore Hospitals for Incurables, 112
" Long Runs," 135
Lying-in Hospital at Helsingfors, 335
Lying-in Hospitals, 207
Madness of the Millionaire, 177
Magic Cures, I., 263; II., 279
Margarine Adulteration, 306
Marriage Restriction, 200
Meath Hospital, 24, 40
Medical History (*continued*) :—
XXVI. The Eclectics and Compilers, 21
XXVII. Byzantine Medicine, 35
XXVIII. Arabic Medicine. (1) The Translators.
XXIX. Arabic Medicine. (2) The Eastern Caliphate, 68
XXX. Arabic Medicine. (3) Haly Abbas and Avicenna, 85
XXXI. Arabic Medicine. (4) The Western Caliphate, 99
XXXII. Arabic Medicine. (5) Hospitals and Institutions, 115
XXXIII. General Survey of Arabic Medicine, 133
XXXIV. The Darkest Age, 147
XXXV. The Darkest Age, 163
XXXVI. The School of Salerno, 179
XXXVII. The Ladies of Salerno, 215
XXXVIII. The Arabo-Scholastic Revival, 245
XXXIX. The Arabo-Scholastic Revival. (2) Surgical, 261
XL. The Close of the Middle Ages, 277
XLI. Mediæval Military Medicine, 293
XLII. The Mediæval Physician, 308
XLIII. The Medical Profession in the Middle Ages, 325

Medical History (continued)—
XLIV. Unprofessional Medicine in the Middle Ages, 341
XLV. Revival of Learning, 357
XLVI. The Mystics, Sympathy, and Signatures, 373
XLVII. Paracelsus, 389
Medical Microscopy, 140
Medical Missionaries, 265
Medical Peers, 87
Medical Protection, 223
Medical Watchmen and Cholera, 181
Mediterranean Winter Resorts, 318
Methods and Morals of Somerset House, 71
Metropolitan Asylums Board, The, 282
Metropolitan Hospital, 40
Metropolitan Hospital Sunday Fund, 152
Metropolitan Workhouses, 8
Middlesex Hospital, The, 160, 363
Mildmay Medical Mission Hospital, 303
Mildmay Mission Hospital, 72, 353
Milk v. Milk Skimmed, 181
Mind, Matter, Medicine, 101
Miraculous Cures of Lourdes, The, 12
Mirth in a Model Lodging House, 167
Miscellaneous Special Hospitals 208
Mr. Chamberlain on Friendly Workers, 259
Moral Antiseptics 129
More Bacteria, 393
More Cholera Precautions, 377
Morningside Asylum, Edinburgh, 61
Musical Amnesia, 231
Napthalin as a Sedative, 294
National Hospital for Paralysis, 8
National Life Boat Institution, 410
National Society for the Employment of Epileptics, 266, 298
Necessity of Frivolity, 182
New General Hospital, Birmingham, 88, 182
New Hospital for Women, 120
New Medicines for New Times, 97
No Quarantine, 278
Nor Friends, Nor Money, 217
North-Eastern Hospital, 40
Nottingham General Hospital, 152
Nurture of Royal Infants, The, 195
On Amputation, 74
One Merry Christmas Bell, 193
Ophthalmia at Hanwell, 181
Opium, Abuse of, 100
Oral Teaching College, 136
"Ordeal of Pauperism," The, 130
Oresund Hospital at Copenhagen, 32
Orthopædy, 414
Other Side, The, 228
Outpost Hospitals in Large Towns, 399
Overstrained Brains, 377
Oxford at its Best, 199
Oxford Doings Extraordinary, 103
*Pall Mall Gazette* Inside the Hospitals, 23
Papier Maché Hospital, 376
Parson and Public house, The, 327
Pasteur, M., 234
Pasteur and Jenner, 262, 265
Pasteur's Seventieth Birthday, 217
Patent Medicines, 248
"Patients Conscientiously Treated," 409
Paying Patients at Special Hospitals, 176
"Penny Dreadfuls," 330
Pensions for Old Age:—
I. Three Schemes Considered, 374
II. Essential Points, 390
People's Needs, The 225
Pernicious Street Begging, 72
Perry, Dr. Edwin C., 200
Personalities, 82
Physics, Lenten Pastoral, 339
Physiology Made Easy, 232
Pictorial Medicine, 124
Plea for the Practical Man, A, 17
Plumbing and Cholera, 329
Poor London, 162
Poor of London and their Medical Needs, 417
Poplar Hospital Accidents, 250
Practical Departments:—
The Laundry, 14; I. Children's Cots, 79; II. Children's Cots, 94; III. Children's Cots, 110; I. Bedsteads, 158, 191; I. Cooking by Gas, 239; II. Cooking by Gas, 255; III. Cooking by Gas, 272; IV. Cooking by Gas, 287; Lockers, 368; Hospital Dresser's Table, 400; Kitchen Ranges and Stoves, 418
Prehistoric Trepanning, 342
Prevention of Consumption 355
Prevention of Disease in Ceylon, 344
Progress in Bacteriology 342
Progress of English Nursing, 401, 416
Progress of Hygiene, 366
Public School Boy's Body, The, 1
Public School Boys, 52
Quain's Elements of Anatomy, 382
Radcliffe Infirmary, Oxford, 104, 280, 314, 362, 369, 410, 415
Railway Servants' Vision, 161
Rates, 358
Rates v. Subscriptions, 66
Recognition of Scientific Research, 294
Richmond Hospital, 48
Ring Out the Old, 217
Riotous Living at Kidderminster Workhouse, 135
Roberts, Mr. G. E., 24
Rotunda Hospital, 120
Rouge, 168
Royal Albert Edward Infirmary, 85, 274
Royal Eye Hospital, Southwark, 175
Royal Eye Hospital, Southwark, Opening of, 258
Royal Frederick Hospital, Copenhagen, 80
Royal Infirmary, Dundee, 239
Royal Infirmary, Liverpool, 314
Royal Infirmary, Newcastle, 314
Royal Medical Benevolent College, 306
Royal Sea Bathing Infirmary, 168, 357
Royal Westminster Opthalmic Hospital, 394
St. George's Hospital, 120
St. Leonard's Hospital, Bedford, 234
St. Mary's Hospital, Clarence Memorial Wing, 194
St. Mary's Hospital, New Wing, 88
Salisbury Infirmary, 56, 72
Salisbury, Marquis of, at Oxford, 371
Samaritan Societies, 294
Scarlet Fever, 56
School Girls, Food and Work, 307
Schoolmaster Very Much Abroad, 98
Seaman's Hospital, 354
Seamy Side of Home, Sweet Home, 409
Secret of Secrets, 233
See Saw, 167
Shall Doctors Take the Lead? 7
Sheffield General Infirmary, 109, 126
Singing Masters' Science, 7
Skin Grafting, 248
Smith, W. H., "Isolation Hospital," 62
"Snowball" Cases, 40
Salt v. Hard Water, 281
Special Hospitals, Lying-in, and Women, 31
Special Hospitals—Children, 13
Special Hospitalsp v.Othalmic & Miscellaneous, 45
Spinal Caries in Sick Children, 316
Steele, J. C., Dr., 111, 140
Stockton Hospital, 48
Story of Insane from Year to Year, 273, 336, 352
Strength, 361
Sunday Fund Meeting, The, 146
Sunderland Infirmary, 319
Sweet Charity's Guide for Christmas Givers (Christmas Number)—
General Hospitals, 203
Special Hospitals, 208
A Few Other Charities, 209
Taken in Trust, 244
Tait, Mr. L., and Vivisection, 370
Tennyson's, Lord, Kindness to Nurses, 39
Testimonial to Dr. M'Cash, Dundee, 234
The Beginning of Disease, 291
The Doctor Day by Day, 361
The Doctor, the Workshop, and the State,
The Drunkard's Deadlock, 249
The Enthusiasm of Medicine, 377
The Indispensable "Spade," 249
The Last New Thing in Hospitals, 114
The Laundry, 14
The Practical Man, 71
The Use of Spemina, 262
The Year-Book of Treatment, 350
This Above All, 211
Tibbitt's, Dr., Libel Action, 345
Too Few Frenchmen, 262
To Will and to Do, 50
Trained Almoners, 217
Trained Nurses and Private Patients, 151
University College, Bristol, New Wing, 14
Value of R.I.B.A., The, 119
Varicose Veins, 73
Vegetarian Society, London, 330
Vibrating Helmet, 86
Victoria Hospital, Folkestone, 174
Virchow Banquet, The, 405
Vivisection: An Appeal to Reason, 92
Vivisection at Church Congress, 39
Vivisection, The Fruits of, 118
Vivisection, The Morality of, 92
Vote of Confidence in Modern Science, 151
Warneford Hospital, Leamington, 256
Warner, Miss, and Dr. L. Tait, 192, 210
Waste Products, 340
Water Supply and Waste, 247
Weak Minded People, 234
What is the Pension Fund Doing? 387
What Shall we Do with Our Dead? 249
What to do with Female Drunkards, 39
Where are the Donors? 34
Where are the Glasgow Newspapers? 103
Where to Send Christmas Boxes, 210
Wholesome Bread, 140
Why do we Shiver? 310
Why is the Hospital the Hospital? 71
Winters, Ancient and Modern, 233
Wisdom of Moderns, The, 49
Within the Hospitals:—
Aberdeen and Its Institutions, 29; Devon and Exeter Hospitals, 46; Morningside Asylum, Edinburgh, 61; Leeds General Infirmary, 77; Sheffield General Infirmary, 109; Berrywood Asylum, 125; Bolingbrook House Pay Hospital, 157; Edinburgh Royal Infirmary, 173; Bradford Infirmary, 189; Royal Infirmary, Dundee, 239; General Infirmary, Northampton, 271; Bedford and Its Medical Institutions, 303; Sunderland Infirmary, 319; Huddersfield Infirmary and the Durham County Hospital, 335; Cumberland Infirmary, Carlisle, 351; Hull Royal Infirmary, 367; Children's Hospital, Great Ormond Street, 383
Women Hospitals, 208

### THE SPECIAL NURSING SUPPLEMENT (MIRROR).

Accommodations for Accidents, cxci

A Cottage Hospital, clxv

A Critic, cxv

A Friend's Picture, cvii

A Happy Christmas, xcv

Airings for Young Patients, cxxvii

A Lasting Memorial, cxxii

American Addresses, cxxvii

American Diets, cxlv

American Experiences, clxi

American Jottings, xxv

American Methods, cxxxviii

American Notes, lxxxix

American Nurses, clvii

American Training School, An, clxiii

A Modest Request, clxxxi

Anderson, Dr. James, clxxiii

An Acceptable Gift, cxxxv

An Apology, xcv

Anonymous Correspondents, xix

An Australian Nurses' Home, cxv

A New Departure, lxxxv

An Infallible Cough Mixture, cxcviii

An Out-patient Department, vii

An Ungrateful Convalescent, vii

A Plea for the Dolls, clvii

A Pleasant Lecture, xxix

A Pleasant Presentation, cxxxv

A Promising Association, clvii

Arbroath District Nursing Association, ci

A Reasonable Request, cxxi

"Art of Massage," The, xxii

Asylum Attendants (see Lectures for)

Asylum Nursing, c xxvi

At Home by the Sea, c xxiii

A Tribute, lxxxix

A Welsh Minister's Curse, cxv

A Wise Memorial, cvii

Bangles at Bedsides. xix

Bantry Board and its Fever Nurse, xiii

Banished Beds, clxxxi

Bath Royal United Hospital Private Nurses, xix

Battenberg, Princess Henry of, clxxiii

Bazaars and Benevolence, i

Bedsores, cxxxi

Belfast Nurses' Home and Training School, lxxxiii

Bitterness, lxxxvii

Bolton District Nurses' Association, xix

Bournemouth Poor, cxli

Brabazon Home of Comfort, Reigate, vii

Brassy Home, Ventnor, xvii

Brave Nurses, clvii

Burdens, clxxi

Burne Jones at the New Gallery, clxiv

Caldecot Hospital Convalescent Home, Bushey, xvii

Carbolised Sheet, clxix., clxxvii

Casualties at Chicago, cxci

Charity Think no Evil, cxxiv

Children's Hospitals and Convalescent Homes, vii

Cholera and Common Sense, clxix

Cholera Precautions, cxl

Christmas Competitions, xx

Christmas Festivities, cx., cxviii

Christmas in Hospital, xcviii

Christmas to an Invalid, xcviii

Cigarettes and Cylinders, i

City Asylum, Birmingham, clxxix

Clapham Maternity Hospital, clxxxi

Comfortable Contributions, cxxiv

Comforts in Sickness, cxlix

Common Sense, cxci

Consolation, xli

Consumption, Management of

I. General Description of Consumption, cxliii

II. Symptoms and Signs of Consumption, cli

III. Hæmaptysis, clix

IV. Coughing, clxvii

V. Sweating and Diarrhœa, clxxv

VI. The Temperature, clxxxiii

VIII. Pneumothorax and Ulceration of the Larynx, cxciii

Convalescent from Fever, clxxiii

Co-operation, cxcvi

Co-operation or Competition, cxcvii

Costwold Benefit Nursing Association, ci

Croupous Pneumonia, cliii., clx., cxlvi

Cumberland Infirmary, xcv

Degrees of Training, xxiii

Delightful Assurance, cxlix

Derby County Asylum, clxxxii

413042

Derry City and County Infirmary
Development of Children by Gymnastics:
I. Popular Notions, xc
II. Gymnastcs, cii
III. Dumbell Exercise, cix
IV. Indian Clubs, cvvii
V. cxxiii
VI. Swedish Drill, xxxix
VII. Ling's System, cxxxvii
Difficult Nursing, clxxxi
Disabled Nurse, A, cxxxii, cxi, cxli, cxlv, clxv, clvii, cxlix, clxxxi, clxxxii
Dundee Royal Infirmary, i
Dying, not Drunken, clxix
East-End Mothers' Home, cxci
Economy at Macroon, xiii
Eccentric Instruction clxv
Edinburgh Royal Infirmary, ci
Empress Frederick, The, cxlix
Endowed Bed, cxxiv
English Nurses for San Remo, cxxxv
English Invalids at San Remo, l
England v. America, cliv
Enlargement and Improvement, clxxxi
Enthusiasm at Darlington, cxxvii
Europeans in India, cxxxv
Example, clxiii
Experiences, clxxvii
Faulty Sanitation, cxlix
Festivities in the Emerald Isle, lxi
Fees and Co-operation, lxxxix
Fixed v. Moveable Seats, clxv
Foundlings and Fevers, xxv
"Fragrance," lxvii
From Wash Tub to Ward, clxxxi
Funeral Honours, lxxxiii
"Fuss," xcv
Girl Nurses, cxxiv, cxxxv
Girl Nurses Again, clx.
Girl Nurses' Infirmaries, cxlv
Glasgow Sick Poor Nursing Association, lxxxiii
Good-bye, Christmas, cxxii
Good Health and Good Temper, cxci
Grange Convalescent Hospital, cxxiv
Growth, xxii
Guildford Institute, The, xxxi
Gymnastics (*see* Development of Children by)
Half-hearted Benevolence, cxv
Having or Giving, civ
Health Classes for the People, xlvi
Health Talks, cxci
Helpless Patients, cxxxviii
Help the Nurses to Help the Sick, xcix
Hewer, Mr., The late, cliv
Holiday Homes, cxxxii
Home for the Dying, Friedenheim, xi
Home Missions, clxv
HOSPITAL, THE, Endowed Bed, cxxxi, cxxxv, cxxxviii, cliv, clvii, clxv, clxix, clxxvii, cxli, cxlv, cxlix, clxxxi, cxci
House of Recovery, cxxvii
How to Become a Nurse and How to Succeed, iv
Hull Royal Infirmary, clxxiii
Incurable Children, xxxi
Indian Nursing, cxlv
Infection in Holland, clxxvii
Institute Nurses in Australia, cxxvii
International Nursing Congress, The Chicago, cxix, cxxvii, cxxxiii, cxlv
Mrs. Bedford Fenwick's Position, clxi, clxxix
International Congress of Charities, &c., clxxv
International Nursing at Chicago, cxcii
Interesting and Instructive, clviii
Interesting Presentation, An, v
Jottings from India, lxi
Kindly Encouragement, clvii
"King Lear" at the Lyceum, xlviii
Kingston Board of Guardians, xlix
La Cité de Misère, lx
Lady Doctors, lxi
Lady Doctor as Lecturer, A, cvii
Lady Dufferin's Hospital, Agra, lxxxix
Lectures for Asylum Attendants—
III. Cleanliness, ii
IV. Warmth, viii
V. Food, xiv, xx, xxvi
VI. Some Important Points, &c., xxxii
VII. Management of Patients, xxxviii
VIII. Management of Lunatics, xliv
IX. Epileptics, l
X. Entertainment, lxxxiv
Lectures on Sick Nursing and Economy, xxxix
Leisure Thoughts, cxxv
Liberality at Lancaster, clxxxi
Lincoln Diocesan Conference, xxv
Linen Night Dresses, cxcvi
Little Invalids, cxli
Loaned Loveliness, cxv
London's End, cxxii
London Diocesan Council for Preventive Work, ci
London Obstetric Society, cxliv
London Hospital Training School for Nurses, clxxxv
London Hospital, Whitechapel, clxviii
Long Expected, lxxxix
Lyme Regis, lxxxix
Macclesfield Cottage Hospital and District Nursing, i
Manchester and Salford Sick Poor Nursing Institute, ci
Marsden's, Miss, Book, xliii
Mary Wardell Convalescent Home for Scarlet Fever, xxxii
Massages, Training in, xl
Mater Misercordiæ, Dublin, cxvii
Medico-Psychological Association, cxxv, xcii
Medicine With or Without Nursing, cxxii
Melancholy, xciii
Merit and Modesty, lxxxv
Metropolitan Asylums Board, xlix
Metropolitan District Schools, xi, xxiii
Midleton Guardians and the Nuns, vii
Military Hospitals, cxxx
Mill Road Infirmary, cvii
Mind and Body, cxlix
Moderate Remuneration, cxci
Modern Methods, clvii
More Co operation, clxxvii
More Cots Wanted, cxci
Mrs. or Miss, cxli
Native Whims, cxli
Neat Beds, iii
Nettle, The, iii
New District Nurse, A, cvii
New Hospital, A, clxxiii
News from Nicosia, vii
Night Nursing, clxxxvi
Northampton Institute for Trained Nurses, xlii
Not a Policyholder, cxix
Notes from Australia, xlvi
Nurse and Superintendent, cxci
Nurse for Seaham Harbour, lxxxiii
Nurse Dolls, The, clxxxi
Nurses and Cholera, cxxxix
Nurses' Annuity Fund, clxxiii
Nurses, Asylum, xlvi
Nurses, A Warning to, cxxxiv
Nurses, Belfast, xlix
Nurses, The Best, cliv
Nurses in Canada, clxxvii
Nurse's Career, A, xxxix
Nurses' Christmas Holidays, cxv
Nurses' Co-operation at Clifton, Bristol, i, v
Nurses, Co-operations for, clxxvii
Nurses Co-operation, The, xi, xix, cxxii, cxliv
Nurses, Dundee, The, xxix
Nurses' Fees, lix, xci, civ, cxix
Nurses for India, clxiii
Nurses for Norfolk, cxci
Nurses, Girl, xv
Nurses, Glasgow, Off Duty, xxxvi
Nurses, Good, Wanted, cxxvii, cxlv
Nurses' Holidays, clxix
Nurses' Home, Boston, U.S.A., cxli
Nurses, Home for Cambridge, xlix
Nurses' Home, Chipping, clxxiii
Nurses' Home, Ipswich, xliii
Nurses, Hull District, xxxi
Nurses in Germany, xci
Nurse's Life in Canada, A, cxciv
Nurses, Leeds District, xxxi
Nurses, Male, The Training of, xxvii
Nurses, Melbourne, cxliii
Nurses, Private, Association & Club, Dublin, lxi
Nurses' Provident Fund, Lowestoft, xxxi
Nurses' Uniforms, lxxxv
Nurses at Vancouver, cxli
Nurse's View of Christmas Decorations, A, xxxiii
Nurses, Windsor, xxxvii
Nurses for Winksworth, cxxxv
Nurses in Workhouses, vii
Nurses, Young, clxi
Nursing Association, Littleborough, i
Nursing, Bombay, lix
Nursing Congress, An International, lxi
Nursing, District, Oddfellows and, v
Nursing, District, Association, Jedburgh, xxv
Nursing, Fifty Years' of, cxiii
Nursing, History of, clv, clxx, cxlviii
Nursing Homes (*continued*)—
VI. Guy's Hospital, x
VII. Birmingham Workhouse Infirmary, xiv
VIII. Royal Free Hospital, ciii
Nursing, Household, xlv
Nursing in India, xlix
Nursing Institute, Croydon, xiii
Nursing in the Land of Ophir, xcvi
Nursing for Natives, clxv
"Nursing Notes" and its Cholera Number, i
Nursing and Nursed, clxxiii
Nursing and Nurses' Institutions in Glasgow, cxxvi
Nursing in Paris, clxv
Nursing, Parochial, in Glasgow, clxvii
Nursing in Portugal, cxcv
Nursing Scheme at Gateshead, xcv
Nursing Scheme, A Provident, lxxxix
Nursing School, A New, lxxxix
Nursing at Sydney, cxlix
Nursing at Tunbridge Wells, cxlix
Nursing in Typhoid, cxii
Nursing at Pembury Workhouse, xiii
Nursing Workhouse Infirmary, Association, xl
Odd Pennies, cxci
On Active Service, i
Our Endowed Bed, cxix, cxv
Our Christmas Parcels, lxxxiii, cxli
Our Fishermen, cxci
Our Indian Letter, xxxiv
Our Pattern, ix
Our Patient, clxxix
Outdoor Relief, clxxiii
Patients' Meals, cxxxviii
Pinnacle of Perfection, The, xlix
Pleading for Trained Nurses, cxxii
Pleasant Entertainment, A, cxli
Pleased Patients, cxv
Poor Barker, c
Portable Tank, clxxxvi
Practical Cyclists, clxv
Practical Lecturing, cxci
Practical Progress, cvii
President, cvii
Progress, clxv
Progress at Dundee, cxli
Progress at Londonderry, cxlix
Progress at Preston, xiii
Proposals in Paris, clvii
Provincial Training, clxi
Princess Louise, cvii
Princess Victoria of Schleswig-Holstein, clxxi
Private Patient's Point of View, From a, xxi
Privy Council and Nurses' Registration, lxxxiii
Quality in Quarantine, i
Queen's Letter, The, cvii
Queen's Nurse for Peebles, cxci
Quickening, clxxxvii
Q.V.I.B., Scottish Branch, ci
Reaping, xxxv
Reasonab'e Regulations, c'xiii
Rest and Fresh Air, cvii, cxxii
Registration, The, of Nurses, xxxix
Royal British Nurses' Association. The, xxvi, xxxvii, cxxxiii, clxii
R.B.N.A., The, and the Lords' Committee's Report, xlv
R.B.N.A., The, and the Registration of Nurses, li, lxii
Royal British Nurses' Soirée, lxci
Royal Commission on the Chicago Exhibition, 1893, xxiii
Royal Edinburgh Asylum, Morningside, clxxviii
Royal Free Hospital, clxxi
Royal National Pension Fund for Nurses, xiii, clxxxiii
Royalty at Portsmouth, clvii
St. John's Maternity Home, xlix, lxxxvi
St. Lawrence's Catholic Home, xcvi
St. Luke's Home, Vancouver, ix, xxv, ci
St. Patrick's Home, clxxxi
Sanitary Wood Wool Company, The, lix
Sarah Ackland Home, The, Oxford, xxxi
Scilly Islands, The, lxxxix
Sectarianism, lxxxix
Sense without Syrup, clxxiii
Shetland Hand-knit Goods, lix
Short Items, xix, xxxvii, xliii, lxxxiii, xcv, ci, cxv, cxxvii, cxli, cxlix, clvii, clxv, clxxiii, clxxxi, cxci
Sick and Hungry, clxxxi
Sick and Poor, clxv
Small Patients at Paddington, cvii
Some Australian Experiences, xvi, xxviii, xlii, lxxxvii xciv, cxlviii, clvi, clxxvii
Some Notable Pictures, xxiv
Soothing Syrups, clxxvii, cxcvi
South Molton and its Nurses' Association, xiii
Stagnation at Eastville, clvii
Stains, Miss, The late, cvii
Stepping Stones, cxcvii
Stockton and Thornaby, xxxi
Successful Sale at Shoeburyness, cxv
Sympathy of Christ, The, xxix
The Daily Walk, lxxxvi
"The Death of Œnone," clxxx
The Latest Hospital Authority, civ
The Life of the Ward, clxxxi
The Little Ring, cxli
The Message, cxl
The New Year, cv
The Result of a Chill, vi, xii, xviii, xxiv, xxx
The Star, clv
The Joke, clxxix
The Young Nurse on Herself, clxxvii
Too Late, clxxiii
Toronto General Hospital, xcv
Tottenham Fever Hospital, vii, xix
Trained Nurses' Club, v, lxi
Training, cxlvii
Tyn-y-coed Convalescent Home, vii
Typhoid in Ireland, cvii
Untrained tho' Kindly, cxxii
Unwatched Death-Beds, cxli
Useful Hints Wanted, cliv
Valued Nurses, cxxvii
Victoriahaus fur Krankenpflege, cliii
V.J.I.N. Edinburgh, xvii
Wanted, A Resident Yardman, xxiii
Want of Money, The, xlix
Wardroper, Mrs., cvi., cxxxii, cxlv
Wardroper, Mrs., and Girl Nurses, cxxxii
Weighed in the Scale, xliii
Welcome Gifts, clxxvii
Where to Live, xlvi
Where Some of our Nurses are Spending Christmas, xcvi
Whitechapel Infirmary, xliii
Whose Fault is It? cxxxv
Wise Doncaster, cxxxv
With Empty Hands, cxv
Women's Convalescent Home Association lxxxiii
Worcester Infirmary, xi
Workers Wanted, cxxxv, cxlix
Workhouse Infirmaries, cliv

Printed by W. H. AND L. COLLINGRIDGE, "City Press," 148 and 149, Aldersgate Street, London, E.C.

### Books Received.

Allman and Son.

"Manual of Hygiene," Parts I. and II. By A. Scholfield, M.D.

"Human Physiology." By E. Parkyn, M.A.

"The Human Body." By Owen Lankester.

"Aids to the Injured Sick." By Henry Gall, M.A.

Oliver and Boyd.

"Manual of Surgical Operations." By Joseph Bell, M.D.

Scientific Press.

"How to Become a Nurse." By Honnor Morten.
"The Art of Feeding the Invalid." By a Medical Practitioner and Lady Professor of Cookery.

Hadden and Best.

"Hadden's Pocket Vocabulary of Medical Terms." Second Edition.

Simpkin and Marshall.

"A Primer of the Art of Massage." For Learners. By Dr. Stretch Dowse.

**Notices of Births, Deaths, and Marriages**, are inserted in this column at a charge of 2s. 6d. for an announcement not exceeding four lines.

### NOTICE TO CORRESPONDENTS.

All MS., Letters, Books for Review, and other matters intended for the Editor should be addressed The Editor, The Lodge, Porchester Square, London, W.

The Editor cannot undertake to return rejected M.S., even when accompanied by stamped directed envelope.

**Notice to Advertisers and Subscribers.**

All Advertisements, Orders for Copies of The Hospital, and Business Communications should be addressed to The Publisher, *not to the Editor*, The Hospital, 140, Strand, W.C.

The Cost of Subscription, post free, is as follows:—Per week, 2½d.; per quarter, 2s. 6d.; per half-year, 5s.; per annum, 8s. 6d. Foreign:—Per annum, 10s. 8d.; payable, in all cases, in advance.

**"Burdett's Hospital Annual," 1891—1892.**

Third year of publication. 600 pp. Boards, gilt lettering. A most complete guide to British and Colonial Hospitals, Dispensaries, Nursing and Convalescent Institutions, and Asylums. Price 3s. 6d. Published by The Scientific Press (Limited), 140, Strand, W.C.

**NOTICE.—The Index for Vol. XI. (ending March, 1892) is on sale at the Office of this Journal, price 2½d., post free.**

### SCRAPS AND GLEANINGS.

Michael Green, the father of 22 children, has recently died at Kildysart, aged 101 years.

Dr William MacEwan has been appointed Regius Professor of Surgery in the University of Glasgow, in the room of the late Sir George H. B McLeod.

The sum of £140 was realized by the bazaar held recently in the Lowey Grammar School in aid of the Cottage Hospital. The patients' stall was presided over by Sister Margaret.

From the official reports it appears that last year articulation was taught to 4,245 pupils in American schools for the deaf. In many cases the infirmity dated from birth, and was inherited.

An application has been made to the trustees of the South Infirmary, Cork, to open that institution to lady medical students. The question was raised by a lady student and Professor Hartog supported it.

A temporary ward built of wood and covered with galvanized iron, has been erected at the Delancey Hospital, Cheltenham, at a cost of £150. The ward contains 20 beds, and is used for infectious cases.

The total annual amount received on behalf of the Hospital Saturday Fund now reaches nearly £13,000, of which sum about £8,000 has been collected in the various industrial establishments of the metropolis.

At a meeting of the delegates from the different friendly societies of the Leominster district, it was decided that the money collected on Hospital Sunday should be given towards the formation of a Cottage Hospital for Leominster.

Mr. G. Q. Roberts has been unanimously appointed House Governor at the London Hospital, *vice* Mr. W. J. Nixon, who has resigned after forty-six years' work there. Mr. Roberts will now hold the combined offices of House Governor and Secretary.

Colonel Robert Stedall, one of the oldest justices of the peace for the county of Middlesex, died last week at The Priory, Highgate. He was for many years chairman of the Highgate Local Board, and formerly commanded the Bloomsbury Rifles.

The proposed additions to the Aberdeen City Hospital are the enlargement of the two centre pavilions by sixteen beds each, the re-arrangement and enlargement of the administration block, additions made to the back wing, and other minor alterations.

The Street Collection on Hospital Saturday in Worcester amounted to £132 12s 6½d.; while at Scarborough, £282 7s.; at Hastings, £249 odd; at Grantham, £169 6s. 6d.; at Twickenham, £1[illegible]1 9s. 3¼d.; at Gainsborough, £44 5s. 4½d.; and at Sidcup £34 12s. 7d. was collected.

It is reported that Drs. Acosta and Rossi, of Cuba, have confirmed the popular notion that disease can be propagated by paper money. They collected from two bank notes circulated in that island over 19,000 germs of different kinds, some of which poisoned rats and guinea pigs.

Upwards of 8,769 in-patients and 27,000 out-patients were treated at the Edinburgh Royal Infirmary during the past year. The hospital contains 680 beds and 30 cots. There are 60 beds at the convalescent home, Corstorphine, making a total of 770 beds always ready for the use of patients.

Miss Alice Bacon, of Newhaven, superintends the Dixie Hospital and Training School for Coloured Nurses, and does a good deal of editorial work for the Hampton paper. She has also taught in the Hampton Normal School for Indians and Negroes for nearly ten years without compensation.

Sir J. B. Maple, M.P., who has already presented an Isolation Hospital for Infectious Diseases, at a cost of £4,000, to St. Albans, now proposes to give the town a recreation and cricket ground. The land comprises over 24 acres, and is conveniently situated at no great distance from the centre of the town.

At a meeting of the Working Men's General Committee, held at Hull, it was proposed to make special efforts to raise £1,000 towards the £3,000 required for the alterations and furnishing of the convalescent home, Withernsea. A special committee was formed to be known as "The People's Mizpah" (gift of £1,000) for furnishing re the Royal Infirmary, Reckitt Convalescent Home.

The Committee of the North Lonsdale Hospital report that there have been an unusual number of accidents treated during the past twelve months. The in-patients numbered 230, and the out-patients 1,533. The Secretary stated that steps were being taken to get the working men of the town to increase their contributions.

The Health Commissioners, Sunderland, have decided to secure a "hopper" from the Wear Commissioners on which to erect a small hospital. The committee intend to have the hospital so erected that it can be taken to pieces when done with, and if necessary be brought out again, and at a very small cost be put upon a floating vessel.

The Teachers' Guild of Great Britain and Ireland is forming an Educational Museum on its premises at 74, Gower Street, London, and has already opened one or two sections of the museum to its members. The Drapers' Company have given 50 guineas, and the Clothworkers £50 towards the museum. The Clothworkers have also promised an annual contribution of £10, subject to certain conditions.

An anonymous donor has given £1,000 to the Essex and Colchester Hospital. A gift of £500 has also been made anonymously to the funds to provide a public park for Colchester, and a similar sum has been given by Mr. S F. Hurnard, of Lexden, towards the erection of a public library. Lord Cowper has promised to add another £100. The park will be opened next month by the Lord Mayor of London.

St Anne's Convalescent Home, Bridlington Quay, has accommodation for 135 adults and 30 children. The home is provided with every comfort, billiard and smoke rooms for the men, dolls houses, rocking-horse, and other toys for the children; and in the women's sitting-room is a piano, besides sofas and easy chairs. The chapel seats about 200 people, and is connected with the home by a covered way.

It is stated that a lady returning from Macclesfield to Manchester, sought in vain for a second-class non-smoking compartment. In despair and with profuse apologies she entered one labelled "Smoking." Upon nearing the next station she observed the gentleman in the carriage collect his goods and chattels together and then carefully remove the red label from the window-pane and insert it in the lining of his hat. This of course revealed the secret of her failing to obtain the seat she required.

### THE STUDENT OF MEDICINE.

[All communications for this Supplement should be addressed to The Editor of The Hospital, at 140, Strand, with the words, "Students' Column," written in the left hand top corner of the envelope.]

### APPOINTMENTS.

Ancrum, G. Wayland, M.B., C.M., L.R.C.S., L.R.C.P. Edin., appointed Assistant House Surgeon to the General Infirmary at Gloucester and the Gloucestershire Eye Institution. Berry, James, B.Sc.Lond., F.R.C.S., appointed Surgical Registrar to St. Bartholomew's Hospital. Edge, Frederick, M.D., B.S., B.Sc.Lond., F.R.C.S.Eng., appointed Honorary Acting Gynæcological Surgeon to the Wolverhampton and District Hospital for Women. Griffith, Augustine, M.B.Lond., appointed Second Assistant Medical Officer to the Nottingham Borough Asylum. Heaton, Authur F., M.R.C.S., L.R.C.P., appointed House Surgeon to the Wrexham Infirmary. Jones, Hugh R., M.D., M.B., B.C.Camb., appointed Assistant Surgeon to the Liverpool Infirmary for Children. Macalister, Charles J., M.B., C.M.Edin., appointed Physician to the Liverpool Stanley Hospital. Madden, Thomas More, M.R.C.P.Irl., F.R.C.S.Edin., L.F.P.S.Glas., appointed Consulting Physican to the Children's Hospital, Temple Street, Dublin. Newbolt, G. Palmerston, M.B.Durh., F.R.C.S.Eng., appointed Surgeon to the Liverpool Stanley Hospital. Nicklin, M., M.B.Lond., L.R.C.P.Lond., M.R.C.S.Eng., appointed Honorary Assistant Surgeon to the Wolverhampton and District Hospital for Women. Ogston, Alexander, M.B., M.D.Aberd., appointed Regius Professor of Medicine at the University of Aberdeen. O'Mara, Francis, L.R.C.P., L.R.C.S.Irel., appointed Assistant Medical Officer to the Limerick Lunatic Asylum. Smith, Patrick A., M.B., C.M., F.F.P.S.Glas., appointed Visiting Physician to St. Peter's Seminary, Bearsden. Thornton, George E., M.D.Edin., M.R.C.S., L.R.C.P.Lond., appointed Resident Clinical Assistant to the St. Marylebone Infirmary.

### VACANCIES.

The following vacancies are announced this week, the amount of salary in each case being given after the name of the institution:

### Resident Medical Officers.

Bethlem Hospital, S.E., two Resident Clinical Assistants—Apartments rations, and attendance provided.

Clayton Hospital and Wakefield Dispensary, Wakefield, Junior House Surgeon; unmarried—Honorarium £40 per annum, with board, lodging, and washing.

General Hospital, Birmingham, House Physician—Salary £70 per annum.

Holborn Union, Resident Medical Officer for the Workhouse, Shepherdess Walk, City Road, N.—Salary £200 per annum, with furnished apartments, gas, coal, and washing.

Hospital for Consumption and Diseases of the Chest, House Physician.

Kent and Canterbury Hospital, Assistant House Surgeon; unmarried—Salary £50 per annum, with board and lodging.

Lancashire County Asylum, Rainhill, near Liverpool, Pathologist—Salary £200 per annum, with furnished apartments, board, attendance, and washing.

### Non-Resident Medical Officers.

Bradford Medical Association, Qualified Out-door Assistant—Salary £120 per annum.

City of London Hospital for Diseases of the Chest, Victoria Park, E. Pathologist—Salary 100 guineas per annum.

County Borough of Stockport, Medical Officer of Health for the District of the Borough, and to the Infectious Diseases Hospital, and Surgeon to the Police—Salary £400 per annum.

General Hospital, Birmingham, Pathologist—Salary £120 per annum.

General Hospital, Birmingham, Surgical Casualty Officer—Salary £70 per annum.

Huddersfield Infirmary, Honorary Physician.

Sunderland Corporation, Medical Officer of Health for the District of the Borough and Port—Salary £500 per annum as Medical Officer of the Borough, £20 for the like office of the Port, and £5 as Public Analyst.

Taunton Union, Medical Officer of the Workhouse—Salary £60 per annum, with 10s. extra for midwifery cases.

University of Aberdeen, Six Examiners for Graduation in Medicine Grant of £30 per annum each.

